# Maternal-infant rotavirus-specific antibody kinetics to inform timing of vaccine boosting in Malawi: An observational study

**DOI:** 10.1371/journal.pmed.1004734

**Published:** 2025-09-12

**Authors:** Jonathan Mandolo, Leah Mulira, Martha Moyo, Memory Mvula, Fatima Mtonga, Marc Y. R. Henrion, Willy Wotcheni, Nigel A. Cunliffe, Kayla G. Barnes, Kondwani C. Jambo, Khuzwayo C. Jere

**Affiliations:** 1 Malawi Liverpool Wellcome Research Programme, Blantyre, Malawi; 2 Liverpool School of Tropical Medicine, Liverpool, United Kingdom; 3 Department of Clinical Infection, Microbiology and Immunology, Institute of Infection, Veterinary and Ecological Sciences, University of Liverpool, Liverpool, United Kingdom; 4 Broad Institute of the Massachusetts Institute of Technology (MIT) and Harvard, Cambridge, Massachusetts, United States of America; 5 Kamuzu University of Health Sciences, Blantyre, Malawi; The Hospital for Sick Children, CANADA

## Abstract

**Background:**

Rotavirus vaccine protects against severe rotavirus-related gastroenteritis. Its effectiveness is substantially lower in low- and middle-income countries (LMICs) compared to high-income settings, partly due to interference from maternally derived rotavirus-specific immunoglobulin G (IgG) resulting from high rotavirus burden. These antibodies wane over time, reducing their capacity to inhibit vaccine-induced immune responses, including immunoglobulin A (IgA). We aimed to estimate the optimal window for administering an additional rotavirus vaccine dose, beyond the routine doses given at 6 and 10 weeks of age, to maximise immunogenicity in an LMIC, high-disease-burdened setting.

**Methods and Findings:**

We collected longitudinal serum samples from 84 infants at five time points between January 2021 and October 2023, and cross-sectional serum samples from 798 healthy individuals aged 0–86 years between December 2022 and June 2024. For participants under 18 years of age, a written consent was obtained from parents or guardians, with assent from the child where appropriate, individuals aged 18 years and above provided written informed consent directly. Rotavirus-specific IgG and IgA concentrations were measured using a gold standard enzyme-linked immunosorbent assay (ELISA). Rotavirus gastroenteritis case data were extracted from ongoing surveillance during the same period. All participants were recruited from the Southern Region of Malawi, a rotavirus high-burden setting. We applied linear mixed-effects and generalised linear models with natural splines to assess age-dependent trends in antibody levels. Prior to scheduled Rotarix rotavirus vaccine dose 1, the median maternally derived rotavirus-specific IgG levels were significantly lower in infants who were seropositive following vaccination compared with those who remained seronegative (5,745.0 IU/ml versus 9,689.8 IU/ml; Wilcoxon-test, *p* = 0.015). Infants with the lowest maternal IgG levels were over five times more likely to seroconvert (odds ratio [OR] = 5.8, 95% confidence interval (CI): 1.6–24.2; Chi-square test, *p* = 0.012). An exponential decay model estimated that the median IgG concentration in non-seroconverters crossed the seroconversion-probability threshold at 6.2 months. Population-level analyses revealed IgG concentrations reached their nadir at 8.4 months, coinciding with a peak in severe rotavirus gastroenteritis cases at 9 months. Serum IgA levels peaked at 9 months and were associated with a decline in disease incidence between 9 and 17 months. Key limitations include the small number of non-seroconverting infants (n = 27) who were unexposed during follow-up and the observational study design. These factors may influence interpretation, but the findings nonetheless provide important insights into rotavirus antibody dynamics.

**Conclusions:**

These findings suggest that administering a booster dose between 6 and 8 months of age, when maternal antibody titer is low and severe rotavirus gastroenteritis risk is high, may enhance the immunogenicity and effectiveness of the rotavirus vaccine in LMICs.

## Author summary

**Why was this study done?:**

- Oral rotavirus vaccines are less effective in low- and middle-income countries than in high-income countries.
- Maternal antibodies passed to infants may block the vaccine from working effectively.
- This study aimed to identify the optimal age for a booster Rotarix rotavirus vaccination in Southern Region, Malawi, an LMIC, high-disease-burdened setting to improve its effectiveness.

**What did the researchers do and find?:**

- We measured rotavirus-specific IgG and IgA antibodies in 84 infants across five time points and in 798 healthy individuals at one time point. We extracted rotavirus gastroenteritis data from our surveillance database to examine the pattern in rotavirus-related severe gastroenteritis with age.
- Infants with the lowest maternal IgG levels before vaccination were 5.8 times more likely to respond to the vaccine than those with the highest levels (OR = 5.8, 95% CI: 1.6–24.2, **p* *= 0.012).
- Antibody levels declined to their lowest point at 8.4 months of age, while severe rotavirus gastroenteritis cases peaked at 9 months, indicating a high risk of infection when rotavirus-specific IgG levels are lowest.

**What Do These Findings Mean?:**

- A booster dose of Rotarix between 6 and 8 months of age, when maternal antibodies are low and disease risk is rising, could improve immune response and better protect infants.
- This study had some limitations, including the small number of infants (27) who did not respond to the vaccine and were not exposed to rotavirus during follow-up, and the fact that it was an observational study, so the results should be interpreted with caution.

## Abstract 3

In an observational study, Jonathan Mandolo and colleagues examined levels of maternal and vaccine-elicited antibodies against rotavirus to provide insights into timing of a booster dose of rotavirus vaccine for infants.

## Introduction

Rotavirus remains a major cause of severe gastroenteritis among under-five children worldwide [1]. Before oral rotavirus vaccine (ORV) introduction, over 500,000 deaths in children <5 years old were attributed to rotavirus annually [2]. Most of these deaths occurred in LMICs in sub-Saharan Africa and Southeast Asia [1]. Post-vaccine introduction, the burden of rotavirus disease fell, with rotavirus-associated mortalities estimated to be 128,500 in 2016 of which 80% occurred in LMICs [1,3]. ORV effectiveness is around 90% in high-income countries (HIC) compared to 43% to 66% in LMICs [4,5].

Malawi introduced a live-attenuated oral G1P[8] rotavirus vaccine (Rotarix) into its national immunisation program in 2012 [5,6]. Rotarix introduction in Malawi led to a decline in the number and severity of rotavirus-related gastroenteritis cases [6–8], and shifted the peak of cases from 9 to 12 months [8]. However, the vaccine effectiveness in Malawi has remained suboptimal at 64%, in line with other LMICs [8,9]. Studies have shown that suboptimal ORV effectiveness might be due to interference from the first dose of co-administered oral polio vaccine (OPV), host Fucosyltransferase 2 (FUT2)-dependent histo-blood group antigen (HBGA) expression, pre-vaccination infant’s gut and maternal breast milk microbiota, and reduced vaccine take due to the presence of high levels of maternal-derived antibodies [10–14].

Rotavirus-specific IgA is a proxy correlate of protection (CoP) against rotavirus infection [15–18], and its induction following vaccination is a key indicator of vaccine efficacy [17,19,20]. Moreover, higher IgA levels are associated with reduced incidence of severe rotavirus gastroenteritis [16]. However, maternal-derived rotavirus-specific IgG in infants can neutralise the live attenuated vaccine virus, reducing its ability to replicate and induce the desired immune response [21]. High levels of maternal antibodies at the time of vaccination are associated with a decreased seroconversion rate and lower levels of vaccine-induced antibodies in infants [13,22]. The impact of maternal-derived rotavirus-specific IgG antibodies on ORVs is more pronounced in areas with high rotavirus burden [16,22–24].

Optimising ORV vaccination strategies to enhance immunogenicity and effectiveness in countries with a high disease burden, such as Malawi, remains a priority. Therefore, we undertook a study to estimate the optimal vaccination window to maximise the immunogenicity of the ORV in Malawi. Our study aimed to identify the optimal timing at which maternal rotavirus-specific IgG antibodies diminish sufficiently to permit a more robust immune response in infants receiving the vaccine, thereby mitigating interference from these antibodies.

## Methods

### Study design

This was and observational study that did not have a prospectively registered protocol or pre-specified analysis plan. Analyses were planned after data collection, and any data-driven changes are described transparently in the methods section. The study utilized data from three cohorts: ROTAHOST, SEROSURV, and DIARSURV.

#### Longitudinal ROTAHOST study.

The main aim of the ROTAHOST study is to elucidate host–rotavirus vaccine interactions using multi-omics approaches, including antibody kinetics, to understand the underlying causes of reduced vaccine effectiveness in Malawi. Infants were recruited using convenience sampling during routine immunisation visits at Chilomoni Health Centre facility in Blantyre City, Malawi and were followed from 6 weeks to one year (January 2021 to October 2023) under the ROTAHOST study. Children were recruited at 6 weeks of age before the first dose of the Rotarix. They then received the second dose at 10 weeks of age. These children were then followed up at 14 weeks of age (4 weeks after the second dose of Rotarix), and subsequently at 6 months, 9 months and 12 months of age. Inclusion criteria included healthy infants aged 5–7 weeks, the parent or guardian planned for the infant to receive both doses of Rotarix, and infants had a guardian that could provide informed consent. Written informed consent was obtained from legal guardians prior to their participation in the study. Guardians were provided with detailed information about the study’s objectives, procedures, potential risks, and benefits before consenting. Exclusion criteria included incapacity of guardian to provide consent, an infant with a severe illness, known HIV–positive status, and being malaria positive.

#### Cross-sectional SEROSURV study.

The primary aim of the SEROSURV study is to establish a population-based sero-surveillance platform to monitor immunity against vaccine-preventable diseases and pathogens with pandemic potential. The platform is designed to estimate cumulative population-level immunity arising from natural infection and/or vaccination, as well as estimate disease burden, across both urban and rural settings in Malawi. Asymptomatic, healthy individuals aged 0–86 years were recruited through random community-based sampling in Blantyre City (urban) and Chikwawa District (rural) between December 2022 and June 2024. Inclusion criteria included individuals willing and competent to give informed consent if ≥18 years old or the parent/legal guardian if the participant was < 18 years old and resided in the census area. Exclusion criteria included being a non-resident in the census area and being deemed clinically unsuitable by the survey team (terminally ill). Written informed consent was obtained from the participants/legal guardians of participants prior to their involvement in the study. The participants/guardians were given detailed information about the study’s aims, procedures, potential risks, and benefits before granting their consent.

We used a subset of individuals enrolled in the parent SEROSURV study for this analysis. For under-five children, all participants with a documented date of birth were included in this analysis. Under-1 children were further grouped based on age in months, mirroring the time points used in the longitudinal sampling in the ROTAHOST cohort. For those above 5 years of age, the following age groups were used: 5–9, 10–19, 20–29, 30–39 and 40 + years. We calculated a sample size for the five age groups so that we could systematically sub-sample from the parent study. We assumed a small effect size (Cohen’s f = 0.225) with a significance level of 0.05, and the desired statistical power of 0.80. Based on these parameters, a total sample size of 240 participants was required. The calculation was performed using the pwr.anova.test() function in R. Samples were then randomly selected using the sample() function in R from a list of 3,425 SEROSURV samples. The number of samples for each age group was calculated based on the age group’s proportion to the sample frame, sex and site. A reproducible random seed (set.seed(7)) was set to allow replication of the sampling process. This approach ensured that the selected sample was unbiased and representative of the target population.

#### Cross-sectional DIARSURV study.

Children under the age of five years who presented with acute gastroenteritis (defined as the passage of at least three looser-than-normal stools in a 24-hour period for less than seven days duration) from November 2012 to December 2024 whose mothers/legal guardians consented to participate in this study were enrolled at both inpatient and outpatient departments, Queen Elizabeth Central hospital (QECH), Blantyre, which is the main referral hospital for the southern region of Malawi. Written informed consent was obtained from the legal guardians of participants before they took part in the study. The guardians were given detailed information regarding the study’s objectives, procedures, potential risks, and benefits prior to providing their consent.

### Ethical approval

National Health Science Research Committee (NHSRC) (protocol number 22/04/2899, approved on 15/07/2022) (Lilongwe, Malawi) and the Liverpool School of Tropical Medicine Research Ethics Committee (LSTM REC) (protocol number 22–053, approved on 22/11/2022) (Liverpool, United Kingdom) approved the SEROSURV study, whereas ROTAHOST study was approved by NHSRC (protocol number 20/04/2521, approved on 18/06/2020) and the Harvard School of Public Health (protocol number IRB18–1,257, approved on 16/11/2018) (Boston, USA). DIARSURV study was approved by NHSRC (Protocol number 867, approved on 18/10/2024) and the Research Ethics Committee of the University of Liverpool, Liverpool, UK (Protocol number 000490, approved on 10/04/2024). All procedures were conducted in accordance with the ethical principles outlined in the Declaration of Helsinki (https://www.wma.net/policies-post/wma-declaration-of-helsinki-ethical-principles-for-medical-research-involving-human-subjects/).

### Sample and data collection

In the longitudinal cohort, a baseline whole blood sample (<2ml) was collected at 6 weeks before the first dose of the Rotarix vaccine. A follow-up whole blood sample was collected at 14 weeks of age (4 weeks after the second dose of Rotarix) and subsequently at 6 months, 9 months and 12 months. In the cross-sectional SEROSURV study, a whole blood sample (3–10 ml dependent upon age) was collected at a single time point. In both studies, participant demographic and clinical data were collected using electronic case report forms (CRF) before sample collection. Serum was separated from whole blood and stored at -80^o^C until the time of analysis. In the cross-sectional DIARSURV study, a bulk stool sample (~5 ml) was collected from each suspected rotavirus case during the acute illness, preferably on the day of presentation to hospital. If sample collection failed on presentation, attempts were be made to collect a stool specimen from all possible cases within 48 hours of hospital admission to avoid the detection of nosocomial infection. Stool samples were refrigerated until screening for rotavirus antigen.

#### Rotavirus-specific enzyme-linked Immunosorbent assay (ELISA).

We employed a previously validated rotavirus-specific antibody-sandwich IgA/IgG ELISA (S1 SOP1, S2 SOP2, S1 Data, S2 Data) [25–27]. In summary, an ELISA plate was coated with a capture antibody (rabbit anti-rotavirus IgG, provided by Christian Medical College, Vellore, India). Then after, rotavirus antigens (Bovine G6P [5] strain WC3) which were propagated in MA104 cells and prepared as cell culture lysates) were immobilized on a plate and incubated for an hour. To account for non-specific antigenic binding, mock-infected MA104 cell lysates were incubated under similar conditions. Serially diluted serum samples were then added and left to incubate for an hour, allowing serum rotavirus-specific antibodies to bind with the rotavirus antigens. To finally detect rotavirus-specific IgA or IgG, biotin-conjugated rabbit anti-human IgA (Jackson ImmunoResearch Europe Ltd, UK) (S1 SOP1) or biotinylated goat anti-human IgG (Vector Laboratories, USA) (S2 SOP2) was used followed by an avidin-biotin-peroxidase complex and a peroxidase substrate. To quantify the rotavirus specific-antibodies, net optical density of clinical samples were compared against an international reference standard. The WC3-based antigen used in this assay does not match the Rotarix (G1P [8], 89–12) strain. However, this assay has been widely used in studies evaluating Rotarix immunogenicity across different settings and has demonstrated utility in comparing the rate of seroconversion and rotavirus-specific IgA titres [13].

#### Rotavirus detection.

A 10%–20% stool suspension for each sample was prepared in 1 ml of sample diluent buffer. Group A rotavirus was detected using the Rotaclone enzyme immunoassay kit (Meridian Bioscience, Cincinnati, OH, USA), following the manufacturer’s instructions.

### Data analysis

Statistical analysis and data visualisation were done using the R environment for statistical computing, version 4.1.0 [28]. Linear mixed-effects models (LME) were used to explore the linear relationship between age and antibody levels in the longitudinal samples, with individual participant’s identification numbers as random effect and age as fixed factor [29]. Continuous variables were compared by *t* test for approximately normally distributed and Wilcoxon rank-sum test for non-normally distributed data. Clopper-Pearson interval was used to estimate confidence intervals for binomial proportions [30]. Categorical variables were compared between groups by using Chi-squared tests. The Kruskal-Wallis test was used to determine if there were statistically significant differences in continuous variables between three or more groups of an independent variable. Spearman correlation was used to measure monotonicity between continuous variables. An exponential decay model was used to understand kinetics of maternal derived rotavirus-specific IgG antibodies to determine optimal time for Rotarix booster dose [31,32]. In the longitudinal cohort study, relationship between categorical outcomes and predictor variables was done by binomial logistic regression.

To explore the linear relationship between antibody levels and age in the cross-sectional samples, we used linear models with natural splines with the number and locations of spline knots chosen based on data distribution and Akaike’s Information Criterion (AIC) [29]. To determine the optimal degrees of freedom (df) for modelling IgG and IgA, we fitted natural spline models with df ranging from 1 to 10 and compared them using AIC, Bayesian Information Criterion (BIC), and Likelihood Ratio Tests (LRT). For IgG, AIC had substantial sequential decreases from k = 1 to k = 5, followed by only small incremental changes until k = 10. BIC however reached its lowest value at df = 5 and LRT results were only statistically significant until df = 5. For these reasons we chose df = 5. For IgA, AIC values decreased substantially up to df = 5, with only marginal decreases beyond that point. BIC was lowest at df = 5 and increased at higher df, supporting the selection of this model. LRT showed significant improvement up to df = 6, but no significant differences beyond this, indicating that df = 5 or 6 was optimal for the model. Based on these criteria, df = 5 was selected as the optimal degrees of freedom for both IgG and IgA models, balancing goodness-of-fit, parsimony, and statistical significance [29]. To visualise the pattern in rotavirus severe cases by age in months, smoothed lines were fitted using locally weighted scatterplot smoothing (LOESS).

Rotavirus seropositivity was defined as detection of rotavirus-specific IgA at ≥ 7 IU/ml following 2 doses of Rotarix for those who were seronegative at week 6. This is based on the assay’s validated lower limit of quantification and the maximum observed value in negative control samples. Seroconversion was defined as detection of rotavirus-specific at either IgA ≥ 20 IU/ml post-vaccination for those initially seronegative at 6 weeks or a 4-fold increase in rotavirus-specific IgA for those seropositive at week 6 [13,20,33].

This study is reported as per the Strengthening the Reporting of Observational Studies in Epidemiology (STROBE) guideline (S1 Checklist).

## Results

### Clinical and demographic characteristics

**Fig 1:**
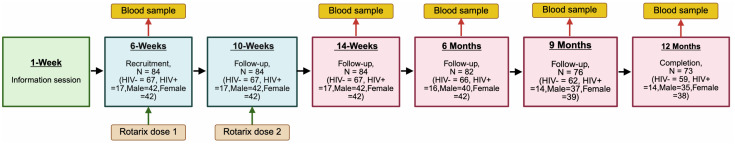
Recruitment and follow-up of participants in a longitudinal cohort study. Participants were recruited at 6 weeks of age and followed at multiple timepoints corresponding to routine immunisation visits, in line with Malawi’s Extended National Immunisation Programme. Blood samples were collected at 6 weeks (baseline), 14 weeks, 6 months, 9 months, and 12 months. Sample sizes and distributions by HIV exposure status and sex are indicated at each time point. HIV- = HIV unexposed infants (born from mothers who were HIV–negative). HIV+ = HIV exposed children (born from mothers who were HIV–positive but on ART).

In the longitudinal cohort, 84 infants were recruited at 6 weeks of age. Out of these, 86.9% (73/84) completed the 12-month follow-up period (Fig 1). Females represented 50.0% (42/84) of the recruited participants, and 20.2% (17/84) of the infants were HIV-exposed (born to mothers known to be HIV–positive but on Antiretroviral Therapy (ART)) (Fig 1). A total of 92.9% ((78/84) children were exclusively breastfeeding and 7.1% (6/84) were mixed feeding. By 6 weeks of age, 4.8% (4/84) of the infants were pre-exposed to rotavirus (rotavirus-specific IgA ≥ 20 IU/ml) before the first dose of Rotarix. The rotavirus IgA seropositivity was 45.0% (36/80; 95% CI, 33.8%–56.5%) for those seronegative at week 6. The seroconversion was 26.2% (22/84; 95% CI, 17.2%–36.9%) post-vaccination.

In the cross-sectional study, 798 persons were randomly selected from the SEROSURV study (Table 1). A total of 49.4% percent (394/798) of the study participants were female, and the median age for the study was 3.0 (interquartile range [IQR] 0.9–6.3) years. Most participants were under-five children (71.1% (567/798)), of which 64.0% (363/567) had no record of Rotarix vaccination, 3.5% (20/567) received a single dose of Rotarix, and 32.5% (184/567) received two doses of Rotarix. The low proportion of children under five years with two confirmed Rotarix doses likely reflects missing or lost vaccination documentation, rather than poor vaccine uptake. As previously reported, rotavirus vaccine coverage in Malawi has been consistently high [34].

**Table 1:** Clinical and demographic characteristics of participants in the cross-sectional study.

Characteristic	Category	Value, N = 798 (%)
**Sex**	Female	394 (49.4)
	Male	404 (50.6)
**Age group**	<3m	46 (5.8)
	3 - 6m	87 (10.9)
	7 - 9m	63(7.9)
	10 - 12m	50 (6.3)
	13 - 23m	76 (9.5)
	2y	82 (10.3)
	3y	90 (11.3)
	4y	64 (8.0)
	5y - 9y	51 (6.4)
	10y -19y	69 (8.6)
	20y - 29y	48 (6.0)
	30y - 39y	29 (3.6)
	40y - above	43 (5.4)
**Rotarix vaccination (<5years)**	No vaccination record	363 (64.0)
	1 dose	20 (3.5)
	2 doses	184 (32.5)
**Site**	Chikwawa	306 (38.3)
	Ndirande	492 (61.7)

*Values are no. (%) except as indicated. IQR, interquartile range. m in the Age group = Months, y = Years

### Association between maternal-derived rotavirus-specific IgG antibody levels and infant Rotarix vaccine antibody response

**Fig 2:**
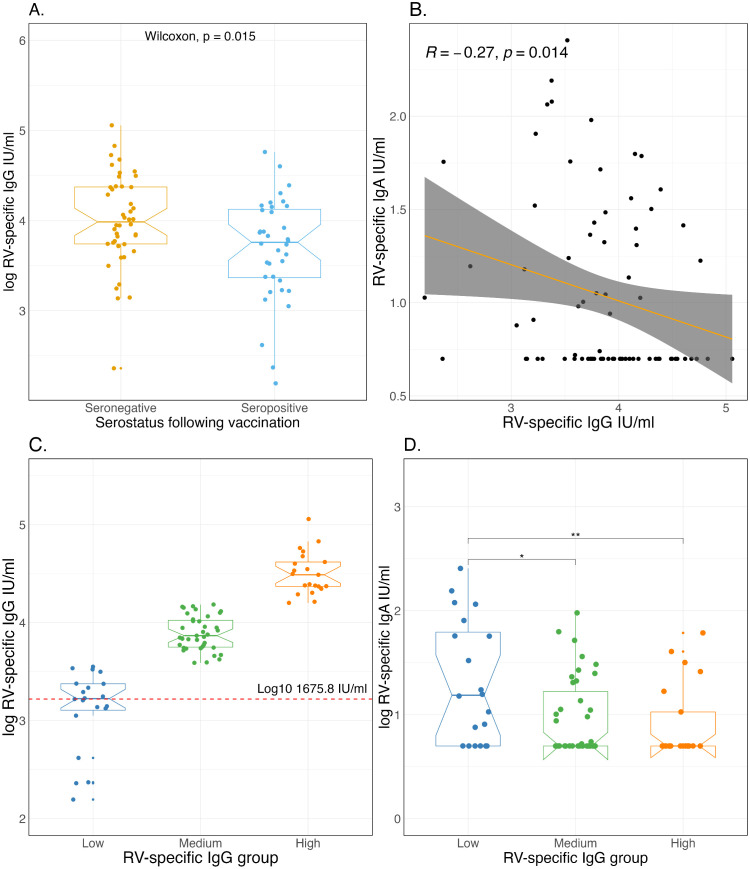
Impact of maternal rotavirus-specific IgG antibody levels on Rotarix response. **(A)** comparison of rotavirus-specific IgG antibody levels at baseline based on rotavirus-specific IgA serostatus at week 14 following 2 doses of Rotarix. **(B)** Relationship between baseline rotavirus-specific IgG levels and week 14 rotavirus-specific IgA levels following Rotarix vaccination. The shaded area in the plot represents the 95% CI around the regression line. **(C)** Week 6 rotavirus-specific IgG groups for children. **(D)** Rotavirus-specific IgA titres at four weeks following second dose of Rotarix based on baseline rotavirus-specific IgG quartile. The red dotted-line on **c** is the median rotavirus-specific IgG (1675.8 IU/ml) at week 6 for infants in the low group. RV, Rotavirus. Asterisks on Fig. D indicate significance levels (p < 0.05 * p < 0.01 **). Box plots show the median (centre line), interquartile range (box limits), and values within 1.5 × IQR (whiskers). Points beyond the whiskers represent outliers. Notches represent an approximate 95% confidence interval for the median.

Maternal-derived rotavirus-specific IgG in infants impacts the ability of the live-attenuated vaccine rotavirus to replicate and induce the desired rotavirus-specific IgA responses [21]. We, therefore, sought to determine a threshold at which maternal rotavirus-specific IgG antibodies diminish sufficiently to permit a more robust immune response in infants receiving rotavirus vaccination. The median rotavirus-specific IgG at 6 weeks was lower in infants who were seropositive following two doses of Rotarix compared to those who remained seronegative (5,745.0 IU/ml ((IQR: 2,323.2–13,350.6) versus 9,689.8 IU/ml ((IQR: 5,511.9–23,616.2); Wilcoxon-test **p* *= 0.015), respectively (Fig 2A). Moreover, there was a negative correlation between rotavirus-specific IgG at 6 weeks, and rotavirus-specific IgA response following Rotarix immunisation (Spearman’s R −0.27, **p* *= 0.014) (Fig 2B).

To assess potential non-linear threshold effects of maternally derived rotavirus-specific IgG on Rotarix vaccine response, infants were stratified into low, medium, and high IgG titer groups based on rotavirus-specific IgG concentrations at 6 weeks of age. Grouping was defined by the distribution quartiles: the low group included infants below the lower quartile, the medium group included those between the lower and upper quartiles, and the high group included those above the upper quartile (Fig 2C). The median IgG concentration in the low group was 1,675.8 IU/ml (IQR: 1,277.9–2,378.5). Infants in the high IgG group mounted significantly lower post-vaccination rotavirus-specific IgA responses compared to those in the low group (Median: 5.0 IU/ml [IQR: 5.0–10.6] versus 15.4 IU/ml [IQR: 5.0–63.0]; Wilcoxon test, *p* = 0.008) (Fig 2D). Infants in the medium IgG group also had reduced IgA responses compared to the low group (Median: 5.0 IU/ml [IQR: 5.0–17.0] versus 15.4 IU/ml [IQR: 5.0–63.0]; Wilcoxon test, *p* = 0.023) (Fig 2D). Given the absence of an established cutoff for maternal IgG interference on rotavirus vaccines, the median IgG concentration of the lowest quartile (1,675.8 IU/ml) was used as a threshold. This provided a data-driven method for exploring potential non-linear threshold effects, while also reflecting the group that exhibited the highest post-vaccination rotavirus-specific IgA response.

Furthermore, we evaluated the association between rotavirus-specific IgA seropositivity and rotavirus-specific IgG groups using logistic regression, with high rotavirus-specific IgG group as the reference category. The participants in the low IgG group had significantly higher odds of IgA positivity compared to those in the high rotavirus-specific IgG group (OR = 5.8, 95% CI: 1.6–24.2; Chi-square test, **p* *= 0.012). In contrast, the odds of IgA positivity did not differ significantly between the medium and high IgG rotavirus-specific groups (OR = 1.7, 95% CI: 0.6–5.8; Chi-square test, **p* *= 0.390), indicating no strong evidence of an association between medium IgG levels and IgA response.

Collectively, the findings demonstrate a significant correlation between maternal-derived rotavirus-specific IgG antibody levels and the subsequent development of infant rotavirus-specific IgA antibody responses post-vaccination.

### Individual-level kinetics of rotavirus-specific antibodies

**Fig 3:**
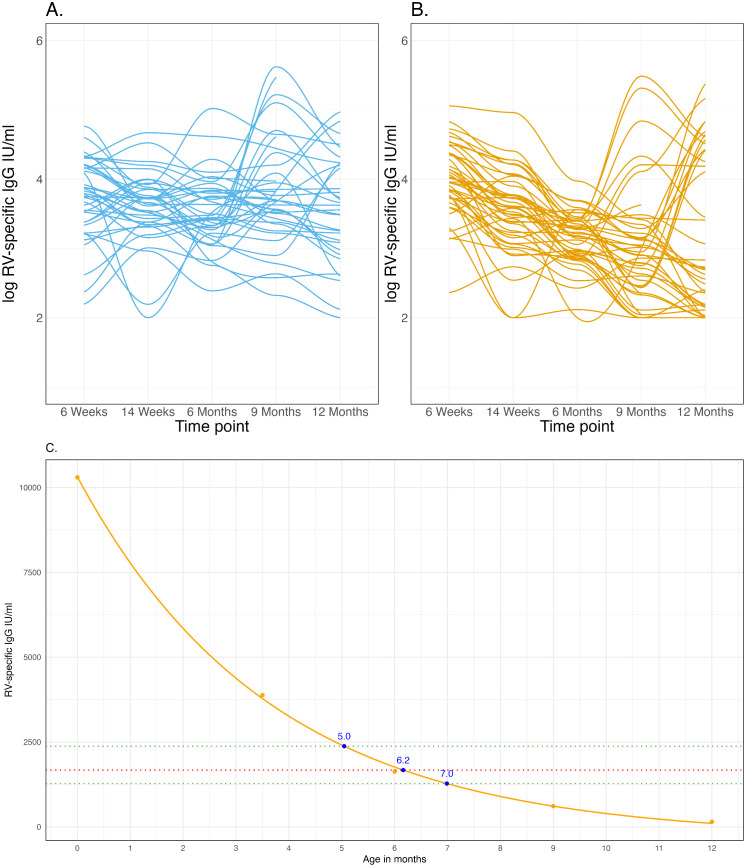
Longitudinal cohort kinetics of rotavirus-specific IgG antibodies. **(A)** Seropositive participants following second dose of Rotarix. **(B)** Seronegative participants following second dose of Rotarix. **(C)** Exponential decay curve for Rotarix seronegative participants who were not serologically exposed after the second dose of Rotarix, defined by absence of ≥3-fold increases in rotavirus-specific IgG titres between any two timepoints. The curved orange line represents the modelled exponential fit, while the orange circular markers indicate the observed median rotavirus-specific IgG levels. The red-dotted horizontal line is median rotavirus-specific IgG (1675.8 IU/ml) at week 6 for infants in the low group and the green-dotted horizontal lines are the corresponding interquartile ranges (1277.9–2378.5) (Fig 2A). RV, Rotavirus. The blue circular markers represent the rotavirus-specific IgG threshold value of 1,675.8 IU/mL and its interquartile range, plotted against age in months.

Next, we assessed the temporal patterns of rotavirus-specific IgG antibodies in this context of high rotavirus burden to identify the period (age) of minimal interference from maternal-derived rotavirus-specific IgG in the first 12 months of life. Overall, we observed at least three distinct temporal patterns of rotavirus-specific IgG antibodies: individuals with a 3-fold increase in antibody levels over time, seroconverters without evidence of waning antibody levels, and non-seroconverters with evidence of waning antibody levels (Fig 3A and 3B). Individuals with a 3-fold increase in antibody levels over time were considered to have experienced a natural exposure event, as previously described [15,35,36]. To determine the optimal window for minimal maternal-derived rotavirus-specific IgG interference and enhanced likelihood of maximal vaccine immunogenicity, we focused on the non-seroconverters with evidence of waning antibody levels (32.1% (27/84)). Utilising an exponential decay model, the median rotavirus-specific IgG among the non-seroconverters surpassed the 1675.8 IU/ml threshold at 6.2 months of age (Fig 3C).

### Population-level kinetics of rotavirus-specific antibodies

**Fig 4:**
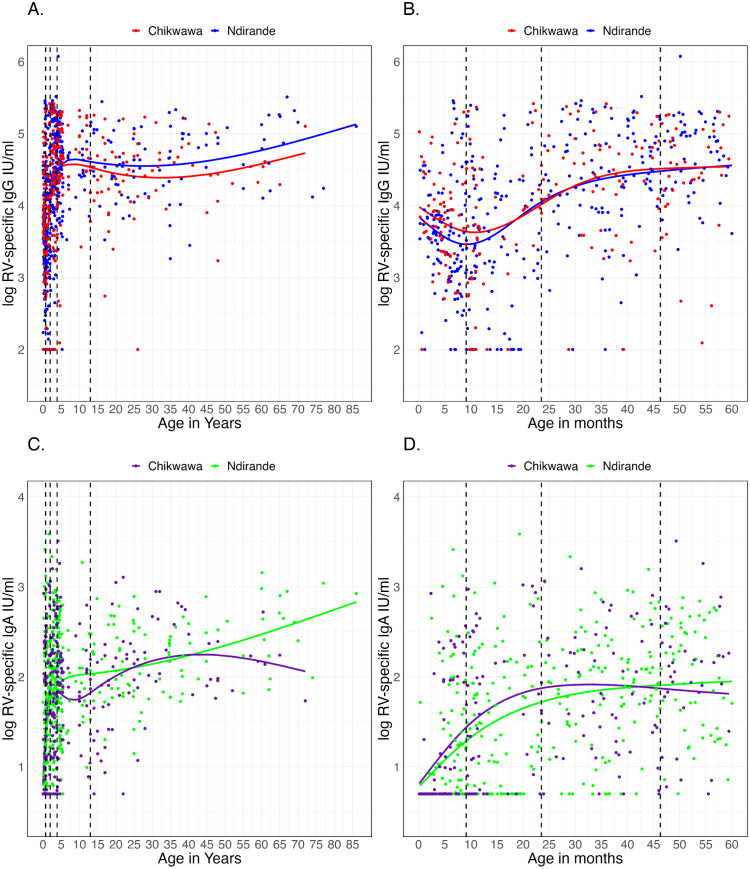
Population level changes in rotavirus-specific IgG and IgA levels by age and site. **(A)** Rotavirus-specific IgG antibody kinetics for all ages. **(B)** Rotavirus-specific IgG kinetics for under 5 years old children only. **(C)** Rotavirus-specific IgA kinetics for all ages. **(D)** Rotavirus-specific IgA kinetics for under 5 years old children only. Dots present actual individual data points. Smooth lines are for predicted values at the population level. Dotted vertical lines are breakpoints from the natural splines. RV, Rotavirus.

We then sought to ascertain the relevance of this optimal window for minimal maternal-derived rotavirus-specific IgG interference at the population level. Linear regression models with natural splines were fitted to examine the nonlinear relationship between age and rotavirus-specific IgG/IgA. Overall, the natural spline model effectively captured the nonlinear trajectory of rotavirus-specific IgG and IgA levels across the life span (Fig 4A–4D). There was a decline in rotavirus-specific IgG levels with age in the early days of life interval up to 0.7 years of age, indicating the waning of maternal-derived rotavirus-specific IgG followed by a gradual increase with age which plateaued by 3.86 years of age (Fig 4A and 4B). In contrast, IgA levels increased steadily after birth, with a sharp rise in early childhood before stabilizing by 1.96 years (Fig 4C and 4D). The data suggest a decline in maternal-derived rotavirus-specific IgG antibodies at a population level during the first 0.7 years (8.4 months) of life, and a parallel increase in rotavirus-specific IgA antibodies. Furthermore, the pattern of rotavirus-specific IgG and IgA antibody levels was similar between those in an urban (Ndirande) compared to rural (Chikwawa) population.

### Comparison of individual and population-level rotavirus-specific antibody seropositivity

**Fig 5:**
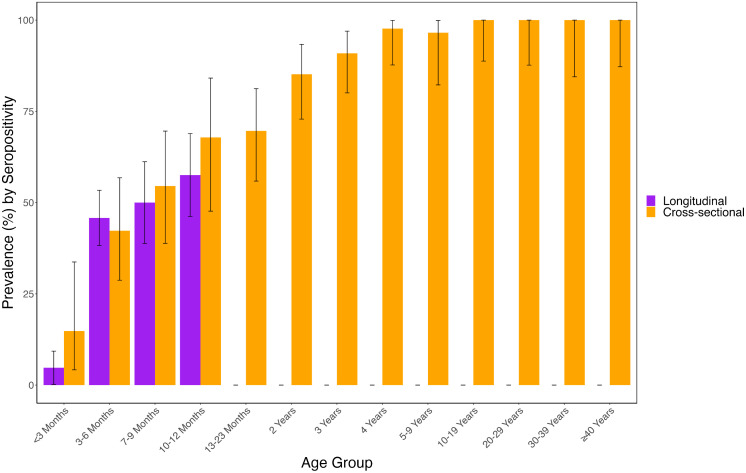
Seropositivity rates by age groups for longitudinal and cross-sectional studies. Seropositivity rates are only based on rotavirus-specific IgA titres. Seropositivity was defined as detection of rotavirus-specific IgA at ≥ 7 IU/ml. RV, Rotavirus. Error bars represent the 95% confidence intervals of the seropositivity rates for each age group.

Next, we compared the individual and population-level seropositivity for rotavirus-specific IgA antibodies. For the population level, only data from Ndirande was used to make the comparison with the individual level cohort to match the geographical settings for the two groups. Overall, there was an increase in seropositivity across age groups during the first year of life at both the individual and population levels (Fig 5). There was no statistically significant difference in the seropositivity at week 6 in the longitudinal cohort compared to the cross-sectional cohort for the < 3 months age group (4.8%, 95% CI: 0.2–9.3 vs.14.8%, 95% CI: 4.2–33.7; chi-squared **p* *= 0.079) (Fig 5), and this was also the case for all the other time points compared to their respective age group (chi-squared **p* *> 0.1). In addition, by 5 years, over 95% of the urban population in the study were seropositive, with 85% by 2 years (Fig 5). Collectively, these results demonstrate a significant alignment between individual and population-level rotavirus immunity and exposure. Furthermore, they suggest substantial exposure to rotavirus during the first two years of life in Malawi.

### Association of population level rotavirus-specific antibody kinetics and severe rotavirus gastroenteritis incidences during the first 5 years of life

**Fig 6:**
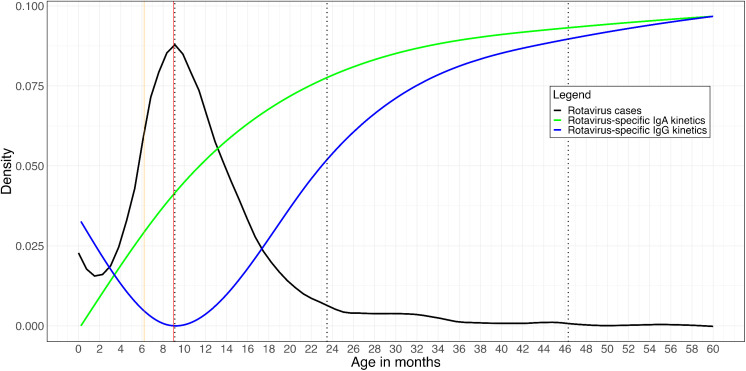
Incidence of rotavirus-related gastroenteritis and rotavirus-specific antibody kinetics in children under five years of age. Severe rotavirus gastroenteritis cases were recruited under the diarrhoea surveillance platform (DIARSURV) at the Queen Elizabeth Central Hospital in Blantyre, Malawi, from December 2022 to June 2024. The rotavirus-specific antibodies were measured in children from Blantyre recruited under a cross-sectional serosurvey in Ndirande from December 2022 to June 2024. The y-axis represents density, defined as the number of rotavirus cases in each age group divided by the total number of cases. The rotavirus-specific IgA/IgG levels have been scaled based on the density values to reflect patterns in case distribution in relation to their kinetics, rather than their absolute levels. Orange vertical line, age in months when the maternal derived antibodies cross the threshold median of 1675.8 IU/ml in longitudinal study. Red vertical line, age in months when most rotavirus cases are recorded. Dotted black vertical lines, breakpoints for both rotavirus-specific IgA and IgG in the cross-sectional serosurvey.

Lastly, we compared the kinetics of rotavirus-specific IgG/IgA antibodies with the incidence of severe rotavirus gastroenteritis cases in under-five children from our ongoing diarrhoea surveillance platform at the Queen Elizabeth Central hospital (QECH), Blantyre. In total, there were 1,199 rotavirus confirmed cases between November 2012 to December 2024. The pattern across age in incidence of severe rotavirus gastroenteritis cases was similar between those collected during the SEROSURV period of December 2022 and June 2024 (n = 122) compared to those not of SEROSURV period (n = 1,077) (Figs 6 and S1). Severe rotavirus gastroenteritis cases increased sharply from 4 months reaching their peak at 9 months of age, and this was paralleled by the decline in maternal rotavirus-specific IgG antibodies at population level from Ndirande (Fig 6). Furthermore, there was a sharp decline in rotavirus-confirmed cases from 9 to 17 months and further decline thereafter plateauing at 24 months, and this was paralleled with a sharp linear increase in population-level rotavirus-specific IgA antibodies in Ndirande reaching their peak at 9 months (Fig 6). In contrast, rotavirus-specific IgG antibodies slowly increased overtime, reaching their peak linear increase at 27 months (Fig 6). Together, these results suggest that the higher incidence of severe rotavirus gastroenteritis in children in Blantyre between 4–9 months of life could be due to suboptimal levels of protective rotavirus-specific antibodies. They also suggest that rotavirus-specific IgG antibodies likely contribute more to protection in the first 6 months of life, but thereafter rotavirus-specific IgA antibodies become essential in protection against severe rotavirus gastroenteritis.

## Discussion

Maternal-derived rotavirus-specific IgG antibodies negatively impact the efficacy of the ORV in infants [13,21,22]. In this study, we aimed to identify the optimal time point when maternal rotavirus-specific IgG antibodies diminish sufficiently to facilitate a robust immune response in vaccinated infants. Our findings revealed diminished response to Rotarix associated with elevated levels of maternal-derived rotavirus-specific IgG in Malawian infants. Our analysis suggested a window around 6 months of age when these maternal rotavirus-specific IgG levels fall below a threshold that could enable enhanced vaccine immunogenicity. The period was also shown to be a window of high burden in severe rotavirus gastroenteritis cases in Malawi.

The low Rotarix seroconversion rate shown in this study is consistent with other studies previously conducted in Malawi and other LMICs [4,13,24,27,37,38]. Similar to other studies, our findings show high maternal antibody levels at vaccination were associated with lower levels of vaccine-induced rotavirus-specific IgA antibodies in infants [13,24]. Those who responded to the vaccine maintained higher levels of both rotavirus-specific IgA and IgG over time, making them less likely to succumb to severe gastroenteritis if exposed to rotavirus [21,22,27]. In contrast, infants with a deficient rotavirus-specific IgA response following vaccination exhibited a gradual decline in their antibody levels over time, reaching a point of diminished neutralising capacity against both natural and vaccine-derived rotavirus strains. Consequently, these susceptible infants are at heightened risk of developing severe gastroenteritis, as supported by the population clinical case-based surveillance data. Therefore, administering an additional dose to these infants could potentially enhance the overall efficacy of the rotavirus vaccine in preventing severe gastroenteritis in this high-risk population affected by rotavirus disease.

Determining the optimal timing for an additional dose of Rotarix requires balancing the waning of maternally derived antibodies, the age-specific epidemiology of rotavirus disease, and the maturation of the infant immune system [37,38]. In this study, we found that infants who did not mount a robust immune response to Rotarix often had rotavirus-specific IgG levels below a threshold associated with improved vaccine immunogenicity at 6–8 months of age. This timing aligns with peak rotavirus infection risk, which occurs between 6 and 24 months of age [38,39], as confirmed by our seroprevalence and case-based surveillance data, and coincides with a period of significant immune system maturation [40]. While delayed vaccination could enhance immunogenicity, it poses programmatic challenges, particularly in settings facing reduced Gavi support and increasing vaccine costs. Nevertheless, optimising vaccine schedules remains crucial to improving effectiveness in low-resource contexts. Clinical trials evaluating an additional Rotarix dose have shown that a third dose at 14 weeks does not significantly improve seroconversion compared to the standard two-dose regimen [19], whereas a booster at 9 months results in higher seroconversion rates [41,42]. A recent meta-analysis further supports enhanced immunogenicity with delayed third doses [43]. However, our data suggest that peak disease burden occurs around 9 months, indicating that this time point may be too late for a booster to confer adequate protection. A 6–8-month window may therefore represent the most strategic timing for a booster Rotarix dose or alternative vaccine, as it aligns with low maternal antibody interference, heightened disease susceptibility, and enhanced immune responsiveness [19,21,22].

Understanding the immunological mechanisms underlying this window of enhanced vaccine responsiveness requires consideration of the central role of mucosal immunity in protection against rotavirus. Gut mucosal immunity plays a central role in protection against rotavirus, an enteric pathogen that infects the intestinal epithelium. Secretory IgA in the intestinal lumen is a key correlate of protection, as it directly neutralises the virus at its site of entry [44,45]. However, due to the challenges of collecting mucosal samples, serum rotavirus-specific IgG and IgA are commonly used as surrogate markers of immunity in clinical studies and vaccine trials [46]. Higher serum IgA titres have been associated with reduced risk of severe rotavirus gastroenteritis [15,18,47]. but the relationship between circulating antibody levels and mucosal immune status remains incompletely understood. Further investigation is needed to clarify the mechanisms of protection and to improve interpretation of vaccine immunogenicity studies. These insights will be critical to refining correlates of protection and evaluating the impact of alternative vaccine schedules or formulations designed to enhance mucosal immunity.

The continued increase in both rotavirus-specific IgG and IgA levels with age highlights the hyperendemicity of rotavirus in Malawi [9]. Rotavirus-specific IgA and IgG levels had a breakpoint around 2 years of age, reaching their peak and maintained thereafter likely by non-severe disease-causing rotavirus exposures in a setting with a high force of infection [39,48]. This finding is consistent with the clinical case-based surveillance in this study, which revealed that severe rotavirus gastroenteritis cases are rarely observed in children after the age of 2. Previous studies have consistently shown that higher levels of these rotavirus antibodies, whether from natural infection or vaccination, are associated with reduced incidence and severity of rotavirus gastroenteritis [15,16,48]. In agreement, we show an alignment between the kinetics of rotavirus-specific antibodies with incidences of severe rotavirus gastroenteritis. A decline in rotavirus-specific IgG in the first 6 months of life associated with an increase in cases, and a rapid linear increase in rotavirus-specific IgA peaking at 9 months of life was associated with a sharp decline in cases. The sustained high levels of rotavirus-specific IgG and IgA levels after 2 years and low number of cases support the notion that natural exposure confers long-term protection against rotavirus gastroenteritis [15,35].

Although this study was conducted in Malawi, the findings of elevated levels of maternal-derived rotavirus-specific IgG interfering with oral rotavirus vaccine (ORV) immunogenicity, suboptimal vaccine-induced IgA responses in early infancy, and an increase in rotavirus-associated infections and seroconversions beyond six months of age, are consistent with patterns reported in other LMICs [13,21,23,24,49,50]. This concordance suggests that the identified window for enhanced vaccine immunogenicity and the potential utility of a booster dose may be broadly relevant across similar high-burden settings.

A major strength of this study is the strong concordance among the longitudinal cohort, cross-sectional serosurvey, and case-based surveillance data. This triangulation supports the conclusion that the kinetics of the rotavirus-specific antibody response at the individual level are mirrored in population-level immunity and may help explain age-related patterns in severe rotavirus gastroenteritis. However, several limitations should be considered. First, although serum rotavirus-specific IgA is associated with protection against severe disease, its absence does not necessarily indicate a lack of immunity. Second, while our analysis identified 6.2 months as the potential optimal window for minimal maternal antibody interference, this estimate is based on a relatively small number of 27 non-seroconverting infants and should therefore be interpreted with caution. Third, although the ELISA used is widely considered a gold standard in vaccine immunogenicity studies, potential antigenic mismatch between the Rotarix vaccine strain and the assay antigen could have led to underestimation of seroconversion rates. Lastly, although our findings suggest that a booster dose administered at 6–8 months may enhance immunogenicity, this may not directly translate into high vaccine effectiveness in settings with intense rotavirus transmission and high force of infection. A clinical trial is warranted to evaluate the impact of an additional rotavirus vaccine dose in such settings and to determine the optimal timing for achieving both immunological and clinical benefit.

Our findings demonstrate a correlation between high maternal rotavirus-specific IgG levels in infants before vaccination and a diminished likelihood of Rotarix immunogenicity in Malawi. A window between 6 and 8 months of age was identified during which maternal rotavirus-specific IgG antibodies reached a threshold associated with an enhanced probability of vaccine-induced rotavirus-specific IgA response in infants within this resource-limited, high-disease-burdened setting. These findings suggest that an additional rotavirus vaccine dose administered between 6 and 8 months could potentially enhance immunogenicity and improve vaccine efficacy.

## Supporting information

S1 SOP1 Laboratory protocol for detection of rotavirus-specific IgA. (DOC)

S2 SOP2 Laboratory protocol for detection of rotavirus-specific IgG. (DOC)

S1 Checklist TRIPODAI checklist. *This checklist is licensed under the Creative Commons Attribution 4.0 International License (CC BY 4.0;* *https://creativecommons.org/licenses/by/4.0/).*
(DOCX)

S1 Fig Trends in Severe rotavirus gastroenteritis cases recruited under the diarrhoea surveillance platform at the Queen Elizabeth Central Hospital in Blantyre, Malawi. Blue smooth curve = Cases registered between November 2012 (when Rotarix vaccine was introduced into Malawi’s national immunization programme) and December 2024. Black smooth curve = Cases registered between December 2022 and June 2024 (a period when SEROSURV study was conducted). Pink smooth curve = Cases registered between November 2012 and December 2024 but excluding those which were collected during SEROSURV period.
(DOCX)

S1 Data Rotavirus-specific IgA and IgG antibody measurements for ROTAHOST study. (XLSX)

S2 Data Rotavirus-specific IgA and IgG antibody measurements for SEROSURV study. (XLSX)
