# Supplementary material for: Maternal-infant rotavirus-specific antibody kinetics to inform timing of vaccine boosting in Malawi: An observational study
Source: PLoS Med. 2025 Sep 12;22(9):e1004734. doi: 10.1371/journal.pmed.1004734 (PMC12445545; doi:10.1371/journal.pmed.1004734)
Supplement: S1 SOP1 — (DOC) [file pmed.1004734.s001.doc]

Title: **Quantitation of Anti-rotavirus IgA by ELISA using 1% blotto Version**

Written by: Dr Khuzwayo C. Jere

Reviewed by: Prof Miren Iturizza-Gomara

Approved by: Dr Kondwani C. Jambo

Effective date: 1^st^ October 2017

# Introduction

Rotavirus is a leading cause of severe gastroenteritis in infants and young children worldwide. Measurement of rotavirus-specific serum and mucosal immunoglobulin A (IgA) is a widely accepted correlate of mucosal immune response and has been used as an endpoint in rotavirus vaccine studies. IgA responses provide valuable insights into both natural infection and vaccine-induced immunity.

# Purpose

The purpose of this SOP is to describe the procedure for measuring and quantifying anti-rotavirus IgA antibodies **measuring anti-rotavirus IgA** antibodies **in human samples**, eg serum or plasma, via **ELISA**. The protocol ensures uniformity and reproducibility across laboratories, enabling comparability of results in clinical and epidemiological studies. It outlines the essential reagents, equipment, and stepwise methodology for the assay, as well as guidance on quality control, data interpretation, and troubleshooting.

The procedure is intended for use by trained laboratory personnel and may be applied in both research and vaccine evaluation settings. Strict adherence to this SOP will promote consistency, reliability, and accuracy of rotavirus IgA measurements across studies.

# Principle of the assay

This assay is based on the sandwich enzyme-linked immunosorbent assay (ELISA) technique. A 96-well ELISA plate is first coated with rabbit anti-rotavirus IgG, then incubated with rotavirus strain (WC3). Clinical test samples with unknown concentrations of anti-rotavirus IgA are added, and quantification is done against a serial dilution of a standard serum/plasma added to each test. Anti-RV IgA antibody detection is done using biotin-conjugated rabbit anti-human IgA antibodies, followed by an avidin-biotin-peroxidase complex and a peroxidase substrate.

Assay controls include high, medium, low positive and negative sera.

The quantity of the anti-rotavirus IgA is determined by comparison of the net optical density of the test samples to the standard curve.

Dilution corrected titers are reported for the test samples if results do not fall within the quantifiable range of the standard curve.

# Procedure

## Materials needed

| Item |
| --- |
| **Equipment**   - Refridgerator (4-8°C) - Biotek Microplate Washer - Shaking incubator (37°C) - Biotek microplate reader and PC - Fine balance - Ph Meter (ensure calibrated) - Multichannel pipette (30-300ul), P0.1-10ul, P20, P200, P1000 pipettes - 1 litre, 500ml autoclaved glass bottles |
| **Plastic Consumables**   - 10ul, 20ul, 200ul, 1000ul non-filter pipette tips - Reagent reservoirs (can be labelled and reused after washing) - Corning Costar 96 well ELISA plate - Corning Costar Plate Lids - 50ml centrifuge tubes - 15ml centrifuge tubes |
| **Reagents**   - Citric acid monohydrate - Sodium Phosphate Dibasic (Na_2_HPO_4_) - Carbonate Bicarbonate capsules - Phosphate Buffered Saline tablets - Tween 20 - Avidin-biotin peroxidase (Vectastain) - O-Phenylenediamine dihydrochloride **15mg** tablets - 30% Hydrogen Peroxide solution - Sulphuric Acid |

## Storage conditions

MA104 and WC3 lysates should be stored at -80°C until use. Once defrosted, lysates can be kept at -20°C for short periods of use. Standard plasma samples and controls and antibodies should be aliquoted in small volumes and store at -80°C until use. Once defrosted, they should be kept refrigerated at 4-8°C while in frequent use.

OPD, ABP and Hydrogen Peroxide should be kept refrigerated at 4-8°C.

Dry reagents for buffers should be stored at room temperature in a cool, dry place.

## Test procedure

### Preparation of Solutions

**Carbonate Bicarbonate (0.05M NaHCO3/NaCO3, ph 9.6±0.2) coating buffer.**

Dissolve contents of 1 Carbonate Bicarbonate capsule per 100ml ddH_2_O. Note the plastic capsule **does not dissolve** – capsules must be carefully opened and contents added. Larger volumes can be made (eg 5 capsules/500ml), autoclaved and stored at 4-8C for up to 2 months. Check pH when making new solution and weekly when stored.

**Wash Buffer (1xPBS-T)**

Dissolve 1 tablet of PBS/100ml (10 tablets/litre) of distilled deionized water (ddH_2_O). (NB PBS tablets may vary in size – always check the bottle for number of tablets/100ml). Add 0.1ml/100ml (0.1% v/v) of Tween-20 (1ml/litre). Make at least 2 x1 litre bottles of Wash Buffer per day. Can be stored if necessary, at 2-8°C for up to 2 months.

**Blocking/Dilution Solution (1%Blotto)**

Dissolve 0.5g of skimmed milk powder in 50ml PBS-T wash buffer. **Prepare fresh before use.**

**Citric Acid Phosphate buffer (0.1M) pH 5.0±0.2**

Dissolve 7.23g of citric acid monohydrate and 9.46g sodium phosphate dibasic in 800ml ddH_2_O. Check pH. Adjust volume to 1 litre with ddH_2_O.

If pH is too acidic, adjust with sodium hydroxide. Make sodium hydroxide solution 12g in 100ml. This solution will become hot so prepare in a cool water bath, in a pyrex bottle, stirring constantly. Add a few drops of sodium hydroxide solution to the buffer at a time, checking pH frequently until in desired range.

**Stop Buffer: 1M Sulphuric Acid**

See separate methods for preparation of Sulphuric Acid. 1M solution can be stored at room temperature for up to 2 months.

### Procedure: Day 1 (3 test plates)

1. Prepare 1:10000 dilution of rabbit anti-rotavirus IgG (3µl of rabbit anti-rotavirus IgG pool in 30ml of carbonate bicarbonate coating buffer). Add 100µl of diluted rabbit anti-rotavirus IgG to each well of each 96-well plate and incubate overnight at 2-8°C. Ensure sufficient volume of lysates and controls required for the next day are removed from archive and in the -20C freezer. Ensure clinical samples are located and transferred to -20C freezer. Ensure there is enough wash buffer for the first wash of the next day.

### Procedure: Day 2 (3 test plates)

1. Prepare 1:4 dilution of MA104 cell lysate (1,875µl of MA104 plus 5625µl of blocking solution, total volume 7.5ml) and 1:4 dilution of WC3 virus lysate (1,875µl of MA104 plus 5625µl of blocking solution, total volume 7.5ml). Note lysates take time to defrost so solutions should be prepared first thing in the morning before plates are removed from incubator.

2. Prime automated washer with PBS-T wash buffer. Wash 5x with PBS-T wash buffer.

3. Add 50µl of the diluted MA104 cell lysate to each well in columns 1, 3, 5, 7, 9 and 11 (virus negative wells). Add 50µl of diluted rotavirus lysate to each well in columns 2, 4, 6, 8, 10 and 12 (virus positive wells). (Follow the Plate Plan).

4. Incubate at 37°C for 60±10min with shaking/rotating (200-270 rpm)

5. Prepare dilution plate during this first incubation. Vortex all clinical samples before diluting. Ensure all initial and serial dilutions are mixed well by pipetting. Use a round bottom dilution plate.

Prepare the highest standard separately (1:160 dilution). Take 5µl standard plasma and add 795µl blocking buffer (total volume 800µl). Add 150µl to A1 and A2 on each test plate (see test plate layout). Add 150µl to the serial dilution plate as follows.

For the IgA standard curve (STD IgA) by making eight 2-fold dilutions of IgA standard (dilutions 1:160, 1:320, 1:640, 1:1280, 1:2560, 1:5120; 1:10240; 1:20480). Take 150mix and transfer 150µl of pre-prepared 1:160 dilution into 150µl of blocking solution (1:320) (well B1 on dilution plate). Mix and transfer 150µl of 1:320 dilution into 150µl of blocking solution (1:640). Continue for further dilutions. Refer to the plate plan and Dilution Plate Layout (Table 1).

For controls, prepare 1:160 dilutions of high (HC), medium (MC) and low (LC) and negative (NC) controls. Make one dilution of each control to use on all 3 plates. Take 2µl of control, add to 318µl of blocking buffer (total volume 320µl). Add 150µl of each control to each of the 3 plates. Refer to the plate plan and Dilution Plate Layout (Table 1).

Prepare a series of four 2-fold dilutions of clinical test samples (TS) (dilutions 1:20, 1:40, 1:80, 1:160). Vortex all samples before diluting. Add 15µl of neat test sample into 285µl of blocking solution (1:20 dilution), mix and transfer 150µl of 1:20 dilution into 150µl of blocking solution (1:40). Mix and transfer 150µl of 1:40 dilution into 150µl of blocking solution (1:80). Mix and transfer 150µl of 1:80 dilution into 150µl of blocking solution (1:160). Refer to the plate plan and Dilution Plate Layout (Table 1).

Table 1: Dilution Plate Layout

|  | 1 | 2 | 3 | 4 | 5 | 6 | 7 | 8 | 9 | 10 | 11 | 12 |
| --- | --- | --- | --- | --- | --- | --- | --- | --- | --- | --- | --- | --- |
| A | Prepare **STD**  **Separately**  **Add 150** µl **to B1** |  | 285µl buffer  15µl  **UK 1** |  | 285µl buffer  15µl  **UK 3** |  | 285µl buffer  15µl  **UK 5** |  | 285µl buffer  15µl  **UK 7** |  | 285µl buffer  15µl  **UK 9** |  |
| B | 150µl buffer |  | 150µl buffer |  | 150µl buffer |  | 150µl buffer |  | 150µl buffer |  | 150µl buffer |  |
| C | 150µl buffer |  | 150µl buffer |  | 150µl buffer |  | 150µl buffer |  | 150µl buffer |  | 150µl buffer |  |
| D | 150µl buffer |  | 150µl buffer |  | 150µl buffer |  | 150µl buffer |  | 150µl buffer |  | 150µl buffer |  |
| E | 150µl buffer |  | 285µl buffer  15µl  **UK 2** |  | 285µl buffer  15µl  **UK 4** |  | 285µl buffer  15µl  **UK 6** |  | 285µl buffer  15µl  **UK 8** |  | **HC**  **Prepare separately** |  |
| F | 150µl buffer |  | 150µl buffer |  | 150µl buffer |  | 150µl buffer |  | 150µl buffer |  | **MC**  **Prepare separately** |  |
| G | 150µl buffer |  | 150µl buffer |  | 150µl buffer |  | 150µl buffer |  | 150µl buffer |  | **LC**  **Prepare separately** |  |
| H | 150µl buffer |  | 150µl buffer |  |  |  | 150µl buffer |  | 150µl buffer |  | **NC**  **Prepare separately** |  |

6. Wash 5x with PBS-T

7. Add 50µl of the IgA standard curve (STD IgA) dilutions, 50µl of the diluted HC, MC, LC and NC and 50µl of diluted test samples to the plate according to the plate plan.

8. Incubate at 37°C for 60±10min with shaking/rotating (200-270rpm)

9. Wash 5x with PBS-T

10. Prepare 1:3000 dilution of biotinylated rabbit anti-human IgA (5µl of biotinylated rabbit anti-human IgA pool in 15ml of blocking solution/PBS); shortly before use. Add 50µl of the diluted biotinylated rabbit anti-human IgA to each well.

11. Incubate at 37°C for 30±10min with shaking/rotating (200-270rpm)

12. Prepare ABP (30 minutes before use). Take 9µl of solution A, mix in 0.5ml eppendorf with 9µl of solution B. Take 15µl this mix and add to 15ml of **PBS-T.** Leave to stand.

13. Wash 5x with PBS-T

14. Add 50µl of the diluted avidin-biotin-peroxidase complex to each well.

15. Incubate at **Room Temperature (RT)** for 30±10min. Shake occasionally to encourage mixing.

16. Prepare OPD substrate (30 minutes before use). Dissolve one 15mg tablet of OPD in 30mls Citric Acid Phosphate buffer, then add 10µl hydrogen peroxide. Leave to stand.

17. Wash 5x with PBS-T, then once with Citric Acid Phosphate buffer (200µl per well).

18. Add 50µl of the OPD substrate to each well

19. Incubate at RT for 30±10min in the dark

20. Stop the reaction by adding 100µl of Sulphuric Acid stop solution to each well

21. Read plates at 490nm using the microplate reader.

# Quality Control, Interpretation of Results, Limitations

After the plate is read Ma104 “blank” values are subtracted from WC3 values and a 4-parameter logistic fit function used to generate final values.

## Validity Criteria

Plate validity if determined by criteria established for the standard curve as well as the controls. Any plates that do not meet the validity criteria have to be retested.

The standard curve is modelled using a four-parameter logistic regression function. The following parameters must be met for the standard curve to be considered valid:

- There must be a minimum of 5 valid points available to generate the curve.
- The Root Mean Squared Error (RMSE) must fall below the acceptable limit.
- The slope must fall within the acceptable range.
- The upper asymptote must fall above the lower limit set for this parameter.
- The lower asymptote must fall below the upper limit set for this parameter.

The controls must fall within the following ranges:

- HC          1124.51-2024.82
- MC         449.67-1009.36
- LC           43.24-205.02
- NC          < 7.00

## Sample acceptance and calculation of titres

For the sample titer to be accepted, the results from two of the four dilutions must fall in the quantifiable range of the curve with a %CV ≤ 20%.

The mean adjusted result is reported if the %CV is ≤ 20%. Masking may be used to obtain a %CV of ≤ 20%. Up to 2 consecutive dilutions may be masked to obtain the desired %CV.

For low titer samples that only have the first dilution within the quantifiable range of the curve, the sample should be retested to confirm the value and that value may be reported.

If all dilutions from one sample fall below the established LOQ, the sample is reported as < LOQ.

## Retesting of Samples

If the above-mentioned acceptance and validity criteria are not obtained, all the samples have to be retested.

If only one dilution falls above LOQ, the sample is retested at the same dilutions to confirm the titer.

If the acceptance criteria still cannot be obtained after retesting the samples once, the sample is then reported as indeterminate.

# Health & Safety

Follow health and safety guidelines as laid down in the MLW Health and Safety Manual.

Discard waste materials in biohazard waste containers.

# Risk assessment Forms

| **SOP for which the Risk Assessment is being done:**  Rotavirus IgA ELISA | |
| --- | --- |
| **Brief description of procedure:**  Sandwich ELISA for anti-rotavirus IgA | |
| **Cadre of staff carrying out the work:**  Laboratory Technicians | |
| **Main Hazard:** | **Precautions required:**  Appropriate use PPE (laboratory coat and gloves) |
| Risk of infection (clinical samples) | Use PPE and universal precautions as outlined in MLW Health&Safety Manual. Autoclave waste products. Wipe up spills with paper towel and place in suitable container. |
| Carbonate Bicarbonate buffer – Irritant. Causes serious eye irritation | Manufacturer recommends eye protection. If in contact with eyes wash with copious amounts of water for at least 15 minutes. Seek medical advice. If in contact with skin wash with soap and plenty of water. If ingested seek medical advice. |
| Phosphate Buffer Solution – Irritant. potential for minor irritation to eyes and skin on contact | As above. In event of skin or eye contact wash with copious amounts of water  If ingested seek medical advice |
| Avidin-biotin-peroxidase, monoclonal antibodies: Non-hazardous | Appropriate use PPE (laboratory coat and gloves) |
| Citric Acid Phosphate buffer**:** Mild irritant | As above. In event of skin or eye contact wash with copious amounts of water  If ingested seek medical advice |
| OPD: Hazardous/Toxic/Carcinogen  Harmful if swallowed or if inhaled  May cause an allergic skin reaction.  Causes serious eye irritation.  Suspected of causing genetic defects. Suspected of causing cancer.  Very toxic to aquatic life with long lasting effects | As above. In event of skin or eye contact wash with copious amounts of water  If ingested rinse mouth, seek medical advice  Do not pour into drains. Dispose of in incineration hazard bag. |
| 1M sulphuric acid – Corrosive. Can cause serious skin and eye damage | As above. In event of skin or eye contact wash with copious amounts of water. If irritation persists seek medical advice.  If ingested seek medical advice |
| **Special risks: not applicable** | **Precautions required:** none |
