# Supplementary figures and images for: Maternal-infant rotavirus-specific antibody kinetics to inform timing of vaccine boosting in Malawi: An observational study
Source: PLoS Med. 2025 Sep 12;22(9):e1004734. doi: 10.1371/journal.pmed.1004734 (PMC12445545; doi:10.1371/journal.pmed.1004734)

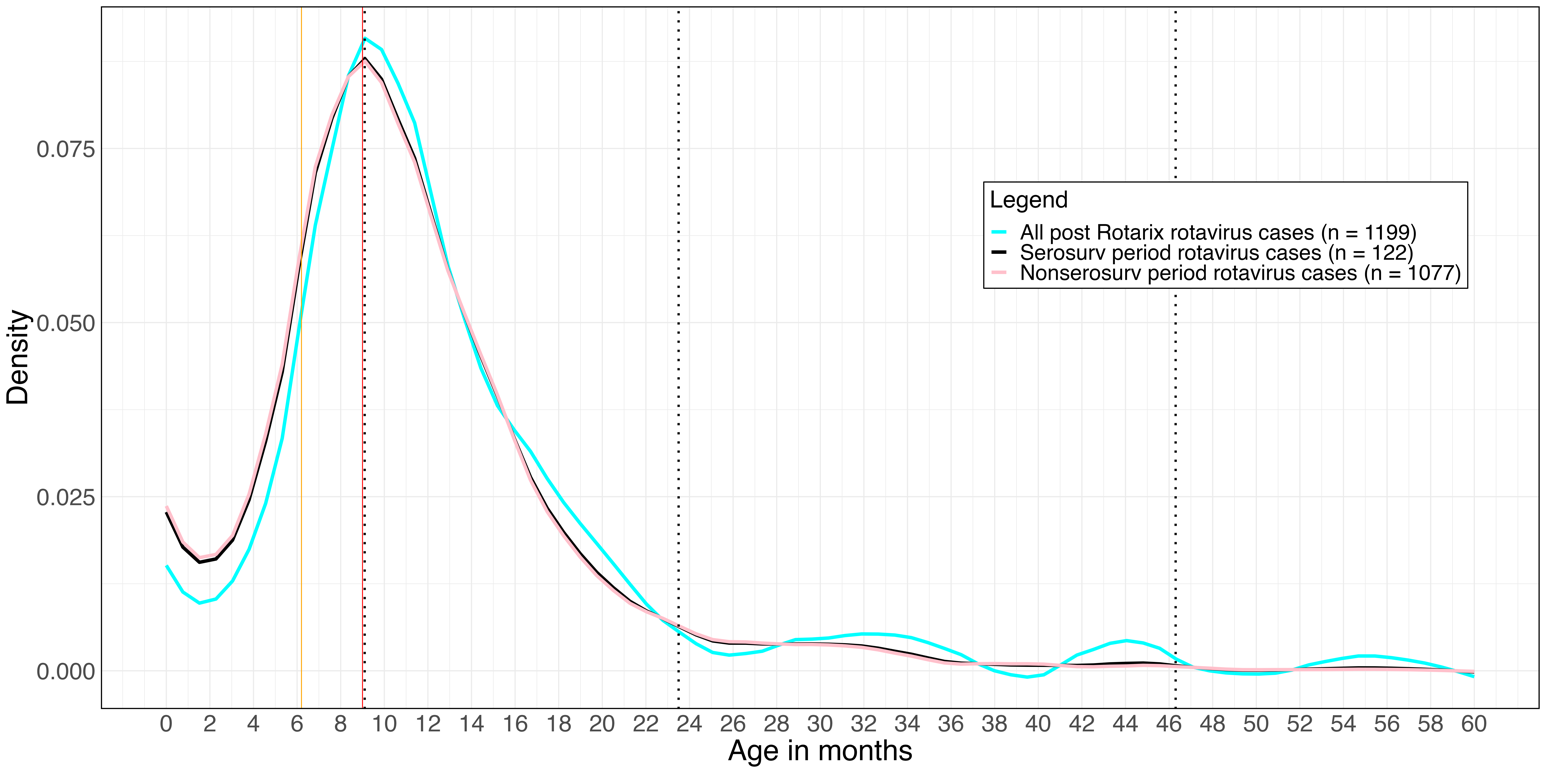

Supplement: S1 Fig — Blue smooth curve = Cases registered between November 2012 (when Rotarix vaccine was introduced into Malawi’s national immunization programme) and December 2024. Black smooth curve = Cases registered between December 2022 and June 2024 (a period when SEROSURV study was conducted). Pink smooth curve = Cases registered between November 2012 and December 2024 but excluding those which were collected during SEROSURV period. (DOCX) [file pmed.1004734.s004.docx]
